# Antioxidant Edible Films Based on Pear Juice and Pregelatinized Cassava Starch: Effect of the Carbohydrate Profile at Different Degrees of Pear Ripeness

**DOI:** 10.3390/polym15214263

**Published:** 2023-10-30

**Authors:** Carmen Rosa Quintero Pimiento, Paula Virginia Fernández, Marina Ciancia, Alex López-Córdoba, Silvia Goyanes, María Alejandra Bertuzzi, María Laura Foresti

**Affiliations:** 1 Universidad de Buenos Aires, Facultad de Ingeniería, Buenos Aires 1127, Argentina; mforesti@fi.uba.ar; 2 CONICET–Universidad de Buenos Aires, Instituto de Tecnología en Polímeros y Nanotecnología (ITPN), Buenos Aires 1127, Argentina; 3 Universidad de Buenos Aires, Facultad de Agronomía, Departamento de Biología Aplicada y Alimentos, Cátedra de Química de Biomoléculas, Buenos Aires 1127, Argentina; vfernand@agro.uba.ar (P.V.F.); ciancia@agro.uba.ar (M.C.); 4 CONICET, Centro de Investigación de Hidrato de Carbono (CIHIDECAR)-CONICET, UBA, Buenos Aires 1428, Argentina; 5 Grupo de Investigación en Bioeconomía y Sostenibilidad Agroalimentaria, Escuela de Administración de Empresas Agropecuarias, Facultad Seccional Duitama, Universidad Pedagógica y Tecnológica de Colombia, Duitama 150461, Colombia; alex.lopez01@uptc.edu.co; 6 Universidad de Buenos Aires, Facultad de Ciencias Exactas y Naturales, Departamento de Física, Buenos Aires 1127, Argentina; goyanes@df.uba.ar; 7 CONICET–Universidad de Buenos Aires, Instituto de Física de Buenos Aires (IFIBA), Buenos Aires 1127, Argentina; 8 Universidad Nacional de Salta, Facultad de Ingeniería, Instituto de Investigaciones para la Industria Química (INIQUI) CONICET, Salta 4400, Argentina; bertuzzi@unsa.edu.ar

**Keywords:** pear juice, pregelatinized cassava starch, non-thermal process, edible films, ripeness degree

## Abstract

Edible films based on fruit and vegetable purees combined with different food-grade biopolymeric binding agents (e.g., pectin, gelatin, starch, sodium alginate) are recognized as interesting packaging materials that benefit from the physical, mechanical, and barrier properties of biopolymers as well as the sensory and nutritional properties of purees. In the current contribution, edible antioxidant films based on pear juice and pregelatinized cassava starch were developed. In particular, the suitability of using pregelatinized cassava starch for the non-thermal production of these novel edible films was evaluated. In addition, the effects on the films’ properties derived from the use of pear juice instead of the complete puree, from the content of juice used, and from the carbohydrate composition associated with the ripening of pears were all studied. The produced films were characterized in terms of their total polyphenol content, water sensitivity, and water barrier, optical, mechanical and antioxidant properties. Results showed that the use of pear juice leads to films with enhanced transparency compared with puree-based films, and that juice concentration and carbohydrate composition associated with the degree of fruit ripeness strongly govern the films’ properties. Furthermore, the addition of pregelatinized cassava starch at room temperature discloses a significant and favorable impact on the cohesiveness, lightness, water resistance, and adhesiveness of the pear-juice-based films, which is mainly attributed to the effective interactions established between the starch macromolecules and the juice components.

## 1. Introduction

In recent years, growing consumer awareness regarding food nutritional quality and safety, individual health, and environmental sustainability has been the driving force behind the search for alternatives to traditional plastic packaging materials [1,2]. In this context, significant progress has been made in the development of biobased and biodegradable materials and products [3]. Polysaccharides and proteins are polymers that address the renewability and biodegradability requirements, and many of them are edible and have good film-forming properties, positioning them as suitable matrices for the production of edible films and coatings [4].

Based on their polysaccharide composition and their distinctive sensory and nutritional properties, fruits and vegetables are interesting ingredients for the production of edible films. Fruits and vegetables, as well as some of their processing by-products and residues, are important sources of nutrients (vitamins and minerals), bioactive compounds (antioxidants, carotenoids, and phenolics), and fiber [5]. Additionally, they also contain polysaccharides and sugars that can function as film-forming agents and plasticizers, respectively [6,7]. Over the past two decades, different films, mainly based on purees and, less frequently, on the extracts and juices of a wide variety of fruits and vegetables as well as their corresponding residues or processing by-products, have been developed [4,8,9]. These films have been proposed not only for coating and packaging applications [10] but also as sweet snacks, including fruit strips, fruit leathers, and fruit rolls. Some of these products have successfully reached the market [11,12].

Even though some reports on the preparation of edible films composed exclusively of fruit and vegetable purees are available, these films have often shown poor consistency and barrier properties, weak mechanical strength, and difficult peeling from the cast surface [4]. Thus, edible binding agents, such as pectin, starch, chitosan, alginate, gelatin, and soy protein, have been commonly added (individually or in combination) to improve the physical properties of films [8]. Additionally, the inclusion of plasticizers (typically glycerol) is a common practice to facilitate processing and to reduce brittleness and stiffness. The incorporation of fillers and nanofillers to enhance the mechanical and physical properties of the films and functional additives intended to provide the packaged product or the packaging material itself with improved sensory, nutritional, and/or microbiological properties have also been widely reported [4,13].

In the current contribution, pears are used as the primary ingredient to produce edible films. Pears are stone fruits produced and consumed all around the world, with an annual global production of 23.1 million tons in 2020 [14]. China is, by far, the largest pear producer, followed by the United States of America, Italy, and Argentina; the latter being the major pear producer and exporter in the Southern hemisphere [15]. Pears are high in fiber, low in calories, and rich in vitamins, minerals, and phytochemicals, especially phenolics, which are important bioactive compounds known for their potential health benefits [16]. Recent contributions have shown that approximately 18% of the losses along the pear supply chain correspond to issues during the harvest phase. These losses are mainly associated with factors such as small fruit size, deformities, and mechanical damage to the fruit [17]. The added-value of these pears, which can be easily collected at producing farms, may be increased by their use in the formulation of edible films. However, to the authors’ knowledge, there is only one early contribution devoted to the preparation of pear puree edible films [18]. In that article, no binding agent was added to the film-forming formulation, and the characterization of the films mainly centered on evaluating water vapor and oxygen permeabilities under different relative humidities and temperatures.

Native granular starch—mainly corn, cassava/tapioca, and potato starch—has been incorporated as a binding agent in different fruit and vegetable puree films. Typically, it has been included at concentrations ranging from 1% to 5%. Starch is a natural polymer composed mainly of amylose and amylopectin chains, whose content varies depending on the botanical source. It is widely abundant, renewable, biocompatible, edible, and biodegradable, and it has relatively low cost, making it an attractive choice. However, the native granular starch is insoluble in cold water, so its use in film formulation requires the disruption of molecular orders within the starch granule through a thermal process known as gelatinization [19]. Granular starch has been used as a binding agent in the preparation of edible films based on mango puree and pineapple pomace [20], carrot puree [21], puree from oil extraction residues [22], blueberry pomace [2,23], papaya puree [24], mango and acerola pulps [25], and mango pulp [26]. In some of the mentioned contributions, the starch was heated to induce gelatinization in the presence of the fruit/vegetable components at temperatures ranging from 70 °C to 90 °C for periods of 10 to 40 min [20,23,24,25,26]. It is worth noting that some reports indicate the potential degradation of phenolic compounds as an effect of the high temperatures used [20]. Alternatively, in other contributions, starch suspensions were first heated to accomplish complete gelatinization, cooled, and then mixed with fruit/vegetable purees to avoid the degradation of their components [2,21,22].

In this context, the use of pregelatinized starches is an interesting alternative for starch incorporation into puree- or juice-based film-forming formulations with no need for heating to induce gelatinization. Pregelatinized starches are pre-cooked starches that can be dissolved in water at temperatures below the gelatinization temperatures [27]. These starches are produced through different techniques, including drum drying, extrusion, and spray drying. In this contribution, pregelatinized starch is used for the first time to produce edible films based on fruits. In particular, pregelatinized cassava starch is specifically chosen as the binding agent for films based on the juice of pears at different degrees of ripeness, which are further characterized in terms of their carbohydrate composition. The selection of this physically modified starch is intended to allow its complete dissolution in the fruit juice at room temperature, preserving the heat-sensitive nutritious components of pears. Additionally, the use of pregelatinized starch facilitates the early and intimate contact of starch macromolecules with the fruit components. Furthermore, the use of pear juice, instead of the complete puree, is aimed at improving the homogeneity and transparency of the films. The produced films are evaluated in terms of their physicochemical and functional properties and compared with films without the addition of pregelatinized starch.

## 2. Materials and Methods

### 2.1. Materials

**Figure 1:**
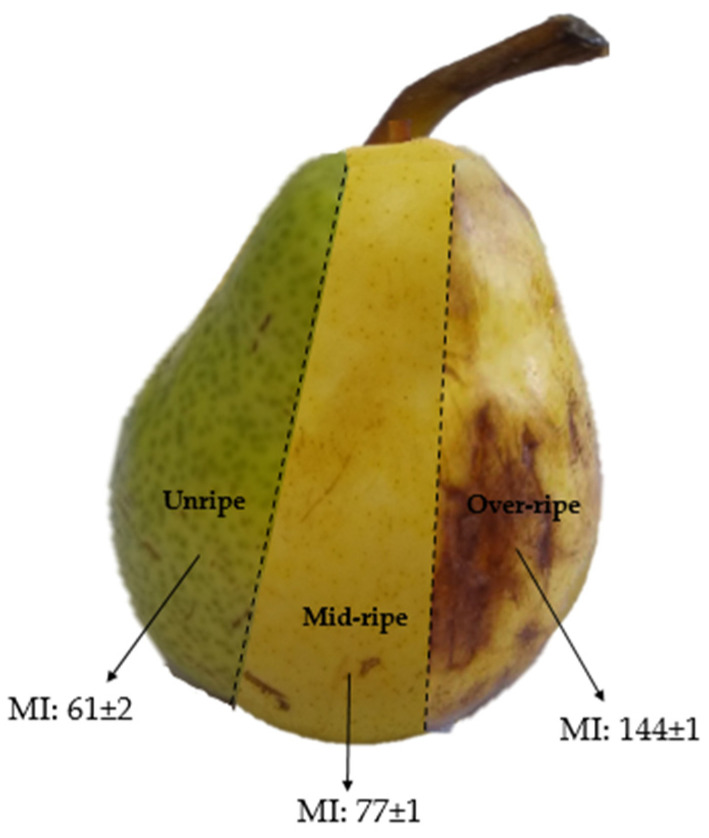
Illustration of pears with the different degrees of ripeness used in this contribution.

William’s pears (11–16% dried matter content (AOAC 335.29)) with varying maturity indices (MI = Soluble solids content (°Brix)/titratable acidity (AOAC 932.12, AOAC 942.15)) were purchased from a local market in Ciudad Autónoma de Buenos Aires, Argentina. Pears were categorized as unripe, mid-ripe, and over-ripe. A representative image of these is shown in Figure 1. Cassava starch was acquired from Cooperativa Agrícola e Industrial San Alberto Limitada (CAISA, Puerto Rico, Misiones, Argentina) and pregelatinized in a laboratory-scale drum dryer under standardized conditions, ensuring a degree of gelatinization exceeding 95% [28]. Glycerol was purchased from Stanton (Ciudad Autónoma de Buenos Aires, Argentina). All other chemicals used were of analytical quality and were used without any additional purification processes.

### 2.2. Obtention of Pear Juice and Purees

Pears were washed thoroughly with tap water, disinfected with 200 ppm chlorine solution for 10 min, and washed in cold water. For the preparation of purees, pears were peeled and cut into quarters, and their core was removed prior to homogenization in a mixer blender (5 min). For the preparation of pear juice, purees were filtered twice using a fabric with a pore size of 239 ± 30 µm.

### 2.3. Determination of Pear Juice Carbohydrate Composition

Pear juice obtained from fruits of different maturity indices was lyophilized. Each lyophilized sample (100 mg) was dissolved in water (20 mg/mL) and dialyzed in a closed system (500 mL) with dialysis tubing of MWCO 3500 Da (Spectrapor, Spectrum Medical Industries Inc., Los Angeles, CA, USA), obtaining one fraction inside the tubing corresponding to the polysaccharides and another one, outside, corresponding to low-molecular-weight compounds. The latter fractions were concentrated and lyophilized, while the former ones were further subfractionated using dialysis tubing of MWCO 12–14,000 Da (Spectrapor, Spectrum Medical Industries Inc., Los Angeles, CA, USA). The original samples, as well as the fractions obtained from fruits of different degrees of ripeness, were analyzed for their total carbohydrate content by the PhOH–H_2_SO_4_ method [29]. The preparation of the samples was carried out in the same manner as for insoluble material [30]. Uronic acid content was estimated using meta-hydroxydiphenyl as the reagent [31]. The monosaccharide composition was determined by the hydrolysis and further derivatization to alditol acetates: the samples were dissolved in TFA 13M (37 °C, 1 h). Then, the acid was diluted to 11.5 M, heated at 100 °C for 1 h, and, finally, the sample was diluted to 2 M to achieve the standard hydrolysis conditions [32]. The hydrolysate was derivatized to the corresponding alditol acetates. In addition, a method comprising reductive hydrolysis and acetylation was performed as a comparison [33]. For gas chromatography of the alditol acetates, an Agilent 7890A gas–liquid chromatograph (Avondale, PA, USA) with a flame ionization detector and a fused silica column (0.25 mm id × 30 m) WCOT-coated with a 0.20 μm film of SP-2330 (Supelco, Bellefonte, PA, USA) was used. The chromatography conditions were as follows: (a) from 200 °C to 230 °C at 1 °C min^−1^, followed by a 30 min hold. Gas chromatography–mass spectrometry of the alditol acetates was carried out on an Agilent 7890A gas–liquid chromatograph with a GCMSQP 5977A mass spectrometer detector (Avondale, PA, USA). Chromatography was carried out as described above, but with He as the carrier gas. Mass spectra were recorded over a mass range of 30–500 amu. For RMN spectra, the sample (10 mg), previously exchanged with deuterium, was dissolved in D_2_O (1 mL) and placed in 5 mm tubes. NMR spectra were recorded at room temperature on a Bruker Avance II 500 spectrometer (Karlsruhe, Germany). Pulse sequences for the ^1^H-^13^C HSQC technique were supplied by the spectrometer manufacturer. Spectra were obtained at a base frequency of 500 MHz for ^1^H and 125 MHz for ^13^C. Signals were referenced to internal acetone (2.21 ppm for ^1^H NMR and 31.1 ppm for ^13^C NMR, respectively).

### 2.4. Preparation of Films

For the preparation of the films, pear puree (26 wt.%) or pear juice (26, 46, 71, and 96 wt.%), glycerol (0.8 wt.%), and distilled water were mixed at room temperature with constant mechanical stirring using a blade impeller (DLab OS40-S, Beijing, China). Then, pregelatinized starch powder (3 wt.%) was slowly added under vigorous stirring. The mixtures were stirred at 1800 rpm for 30 min to obtain homogeneous dispersions, degassed under vacuum to remove bubbles, and finally poured onto leveled Teflon-coated plates. The plates were placed in an oven with air circulation at 30 °C for 48 h. After this period, films (0.30–0.35 mm thick) were peeled off from the casting surface and conditioned at 30% RH at 25 °C for 10 days.

### 2.5. Characterization of Films

The produced films were characterized in terms of their physicochemical, optical, mechanical, barrier, and antioxidant properties. The results are the average of at least three replicates.

#### 2.5.1. Color

The color parameters of the films were determined using a Minolta colorimeter (CR-20, Konica Minolta, Inc., Tokyo, Japan) previously calibrated with a white reflector plate. The films were placed on a white base, and for each sample, ten measurements were taken at random points. Their color was expressed as colorimetric coordinates in the CIELAB scale: L* (lightness, from 0: black to 100: white), a* (from green (−) to red (+)), and b* (from blue (−) to yellow (+)). Color differences (Δ*E*) were calculated using the following equation:

Δ*E* = [(ΔL*)^2^ + (Δa*)^2^ + (Δb*)^2^]^1/2^, (1)

#### 2.5.2. Transparency

The transmittance of the films was measured using a Shimadzu UV/visible spectrophotometer (model UV-1650pc, Tokyo, Japan) at a wavelength of 800 nm. Transparency was determined as the ratio of the logarithm of transmittance (%) to the film thickness (mm) [34].

#### 2.5.3. Moisture Content

The moisture content (%) of the films was determined gravimetrically and calculated as the percentage ratio of the weight loss of the films dried at 105 °C for 24 h to their initial weight.

#### 2.5.4. Water Solubility

To determine the water solubility of the film, samples (2 × 2.5 cm) were dried in an oven at 105 °C for 24 h and then weighed. The dried films were immersed in 50 mL of distilled water for 24 h at room temperature. The insoluble fraction was then carefully recovered, dried again at 105 °C for 24 h, and weighed. The water solubility of the films (%) was calculated as the percentage ratio of the weight loss of the films to their initial dry weight.

#### 2.5.5. Wettability (Contact Angle)

The contact angle of the samples was assessed by the sessile drop method. A drop of distilled water (7 μL) was placed on the sample surface (2 × 2 cm) using a syringe. Measurements (at least three per sample) of the contact angle were performed at room temperature immediately after deposition and also at 150 s after deposition using an Optical Tensiometer (OneAttension theta, Biolin Scientific, Gothenburg, Sweden) coupled with image analysis software (V4.0.3.1338, Analysis Software).

#### 2.5.6. Swelling Degree

To determine the swelling degree of the films, samples (2 × 2.5 cm) were immersed in 50 mL of distilled water for 24 h at room temperature. The insoluble swollen fraction was then carefully recovered, wiped with paper to remove the excess liquid, and weighed (Ws). The recovered samples were then dried at 105 °C for 24 h and weighed (Wd). The swelling degree of the insoluble film fraction was calculated as (Ws − Wd)/Wd.

#### 2.5.7. Water Vapor Permeability

The water vapor permeability (WVP) of the films was determined according to the ASTM E96/E96M-22 standard [35]. Preconditioned samples (50% RH at 21 °C for 2 days) were used to seal the open mouth of circular acrylic test dishes containing CaCl_2_ and kept in a controlled temperature (21 °C) and relative humidity (50%) chamber for 10 days. The dishes were removed from the chamber once a day and carefully weighed in an analytical balance (AS 220. R2, RADWAG, Radom, Poland) to the nearest 0.0001 g. Weight data were plotted as a function of the elapsed time, and the water vapor transmission rate (*WVT*) was determined from the slope of the plot (g/s) divided by the test area (3.8 × 10^−4^ m^2^). Water vapor permeability (*WVP*, g m/(m^2^ s Pa)) was then determined from Equation (2):

(2) $$\mathrm{WVP}=\frac{WVT\;}{S\;\;\left(R1-R2\right)}\;d$$

where *WVT* is the water vapor transmission rate (g/(m^2^s)), *S* is the saturation vapor pressure at the test temperature (Pa), *R*1 − *R*2 is the difference of the relative humidities expressed as fractions (i.e., 0.5), and *d* is the sample thickness (m).

#### 2.5.8. Fourier-Transform Infrared Spectroscopy (FTIR)

Fourier-transform infrared spectra of the films were acquired using an IR Affinity-1 Shimadzu Fourier-transform infrared spectrophotometer equipped with an attenuated total reflectance module (ATR, ZnSe crystal) (Shimadzu, Tokyo, Japan). The spectra of the dried films (70 °C, 2 h, under vacuum) were collected in absorbance mode in the 4000–700 cm^−1^ range with 45 scans at a resolution of 4 cm^−1^.

#### 2.5.9. Mechanical Properties

Uniaxial tensile tests were performed using a Brookfield Texture Analyzer (CT3-100, Middleboro, MA, USA) according to the ASTM-D882-18 standard [36]. The films were cut into strips of 35 mm × 5 mm and conditioned at 56% RH at room temperature for 48 h before being tested at a strain rate of 1 mm/s. The Young’s modulus, tensile strength, and strain at break values were calculated from the stress–strain curves. The reported results are the average of at least 10 replicates.

#### 2.5.10. Total Polyphenol Content and Antioxidant Activity

The total polyphenol content of the samples was measured by the Folin–Ciocalteu method [37,38]. A calibration curve was prepared using gallic acid (Merck, Darmstadt, Germany) as a standard, and the results were expressed as gallic acid equivalents (GAE) per 100 g of sample (mg GAE/100 g).

To determine the antioxidant activity of the films, two established methods were employed according to previously reported protocols: the DPPH radical assay and the ferric-reducing antioxidant power (FRAP) assay, [37,38]. In the DPPH method, a calibration curve was prepared using Trolox as a standard, and the results were expressed as mg of Trolox equivalents per 100 g of sample. Conversely, in the FRAP assay, the calibration curve was prepared with an aqueous solution of ferrous sulphate (FeSO_4_), and the results were expressed as mg FeSO_4_ per 100 g of sample.

#### 2.5.11. Statistical Analysis

R (V4.0.2, Cran Software) and Jamovi (V2.3, The Jamovi project) were used to perform the analysis of variance (ANOVA) and Games–Howell contrast tests. InfoStat software (V2017.1.2) was used to perform the Student’s *t* test. Differences were statistically significant at the *p* < 0.05 level.

## 3. Results and Discussion

### 3.1. Study of the Carbohydrate Composition of Juice from Pears with Different Degrees of Ripeness

It is well known that during maturation, ripening, and senescence, important changes in carbohydrate composition take place. In this context, the carbohydrate composition of the juice from Williams’ pears at different degrees of ripeness (Figure 1) was determined. Pears contain carbohydrates with diverse functions and significantly varying molecular weights, including structural and reserve polysaccharides, monosaccharides, disaccharides, oligosaccharides, and modified sugars such as sorbitol [16]. Thus, three fractions for each ripening stage (i.e., low-, medium-, and high-molecular-weight fractions) were analyzed (Table 1).

It is evident from Table 1 that the low-molecular-weight carbohydrates are the most important in yield, but those with higher molecular weights also contribute significantly. The medium-molecular-weight fractions contain glucose as a major monosaccharide component, which originates from partially degraded starch-derived glucans. Additionally, low-molecular-weight pectins are also evident, comprising around 60% *w*/*w* homogalacturonans (HGs), calculated as (UA + Rha + Ara + Gal − (2×Rha + Ara + Gal)), and 40% *w*/*w* rhamnogalacturonan I (RGI), calculated as (2×Rha + Ara + Gal). For both low- and medium-molecular-weight fractions, there are no linear changes in the amount of different monosaccharides among the pear juices, but the mid-ripe juice shows differences with the unripe and over-ripe samples. Brahem et al. (2017) reported carbohydrate composition values similar to those found in this study for mid-ripe and over-ripe pear pulp [39].

The high-molecular-weight samples are mostly constituted of pectins, comprising HG and RGI. Notably, there is an important difference between those derived from the juice of pears of different degrees of ripeness. The monosaccharide composition clearly shows a reduction in uronic acids and an increase in rhamnose and arabinose contents as pear juices from higher maturity indices are assayed. Upon calculating the mass percentages of both pectin polymers, it is found that the amount of HG decreases, whereas that of RGI increases. Consequently, the overall quantity of pectins remains quite stable (HG: 74, 56, and 30% *w*/*w* and RGI: 26, 44, and 70% *w*/*w*, for unripe, mid-ripe, and over-ripe samples, respectively). These findings imply that HG is degraded during ripening, while the synthesis of RGI is stimulated during this event.

**Figure 2:**
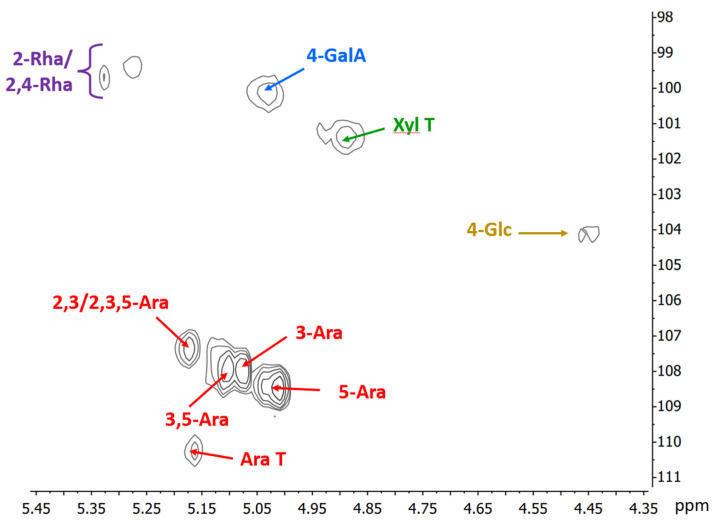
Anomeric region of the HSQC NMR spectrum of the high-molecular-weight sample obtained from the mid-ripe pear juice. Numbers indicate linkage positions; T: terminal unit; Ara: a-L-arabinofuranose; GalA: a-D-galacturonic acid; Xyl: a-D-xylopyranose; Rha: a-L-rhamnopyranose; Glc: β-D-glucopyranose units.

The HSQC NMR spectrum of the mid-ripe pear juice (Figure 2) confirms the results from chemical analyses. The anomeric region shows the presence of the anomeric signal of 4-linked α-D-galacturonic acid at δ 100.2/5.02 and 2-linked α-L-rhamnopyranose units, partially substituted on C4 at δ 99.7/5.33 and 99.4/5.28. Regarding the arabinose side chains of RGI, five important anomeric signals corresponding to different substitution pattern of these units are evident (δ 107.4/5.17 of 2,3-linked or 2,3,5-linked; δ 107.9/07 and 107.9/5.11 due to 3-linked and 3,5-linked; δ 108.5/5.01 due to 5-linked; and δ 110.3/5.16 for terminal arabinofuranose units). Other results from the NMR spectrum show that the galacturonic acids are not methyl esterified, as no peaks at ~53.5–54.0/3.7–3.75 ppm (corresponding to the methyl group of the esterified galacturonic acid) are observed, indicating free negative charges in the pectin chain. In addition, signals at ~21.3/2.05–2.15 ppm correspond to the methyl group of acetyls, possibly linked to C3 of some of the galacturonic acid residues. This is confirmed by the presence of signals at δ 71.7/5.01 and 79.3/4.52, corresponding to C3/H3 and C4/H4 of these units, respectively [40].

In mid-ripe pear juice samples, chemical and spectroscopic analyses reveal the presence of small amounts of xyloglucans and mannans. However, no clear trend regarding their quantities could be discerned. Overall, the results highlight the variation in the composition and molecular weight of the carbohydrates in pear juice during ripening. This variation is expected to significantly affect the properties of the films produced with these juices.

### 3.2. Effect of the Carbohydrate Composition of Pear Juice and the Addition of Pregelatinized Starch on the Handling and Mechanical Properties of Edible Films

Edible film formulations containing the juice from pears at different degrees of ripeness (i.e., different carbohydrate compositions) were evaluated. These formulations were assessed both with and without the addition of pregelatinized starch while maintaining a constant juice content (71 wt.%). It was observed that formulations with unripe pear juice failed to form a film when starch was not added, whereas the over-ripe pear-juice-based film could not be detached from the cast surface. Conversely, the formulation with mid-ripe pear juice produced continuous and detachable films. However, these materials exhibited challenging handling characteristics, including excessive adhesiveness and sensitivity to environmental conditions. These attributes posed difficulties during mechanical testing, particularly in achieving uniform dimensions when cutting strips as well as managing the effect of humidity during testing [41]. The observed variations in the films’ handling characteristics were attributed to the different profiles of low-molecular-weight saccharides found in each juice type (Table 1). Over-ripe pear juice exhibited the highest values of low-molecular-weight carbohydrates (<6–8 kDa), mainly glucose, with values decreasing in the following order: over-ripe > unripe > mid-ripe. Previous contributions have highlighted that films exclusively composed of purees often exhibit poor consistency and mechanical strength, and some may not detach from the cast surface as continuous films [42,43,44]. Consequently, the addition of edible hydrocolloids as binding agents is crucial for enhancing film cohesiveness [4].

On the other hand, when all three juices were combined with pregelatinized cassava starch, the resulting films exhibited good structural integrity, facilitating easy detachment from the cast surface and handling, especially in high-humidity conditions. This observation suggests the generation of effective interactions between starch and juice components. Like other biopolymers such as pectin, sodium alginate, gelatin, and chitosan, starch is frequently included in fruit- and vegetable-puree films. Its addition is attributed to starch’s ability to modify texture, enhance film-forming properties, provide structural integrity, and improve film cohesiveness. Some previous contributions reported that the addition of starch into the film formulation can establish additional hydrogen-bonding interactions between polymer chains, resulting in enhanced mechanical properties [45,46].

Table 2 summarizes the mechanical properties of films containing juice from unripe, mid-ripe, and over-ripe pears. Results evidence that the unripe and over-ripe pear juice-based films exhibit similar Young’s moduli and maximum stress values. However, the film based on unripe pear juice shows a significantly higher deformation at break, surpassing all other films.

On the other hand, the film based on the mid-ripe pear juice shows the highest values for the Young’s modulus and tensile strength, which is indicative of possessing the greatest cohesive energy. This can likely be attributed to the strong interactions between the starch chains and the carbohydrates present in the juice at this ripening stage. In over-ripe pears, pectins are expected to be degraded, generating more open and disordered structures with weaker interactions. These findings align with the discussed variation in the carbohydrate composition of pears (Table 1). As mentioned before, the literature on films made from pear is scarce, and to the best of the authors’ knowledge, there are no previous reports on their mechanical properties for comparison. Beyond the effect of testing conditions (e.g., ambient humidity and temperature, previous sample conditioning, and assay parameters, particularly strain rate) on the mechanical properties of edible films, the literature on films made from fruits and vegetables underscores that these properties are markedly dependent on the film composition [4,8,45]. Considering the substantial changes in fruit composition during ripening as illustrated in Table 1 [39,47,48,49,50], it was expected that the mechanical properties of the films made from their purees or juice would likewise vary with the maturity index of the fruit. This analysis, to the best of the authors’ knowledge, has not been previously conducted for pears. It reveals the strong correlation between the properties of juice-based films with the pear ripeness level, providing a basis for well-founded decisions on the potential use of pear losses and wastes, depending on their degree of ripeness. Despite variations in the testing conditions used, focusing on films made from fruit pulp and starch, the values presented in Table 2 align with those previously documented [4,26,51]. For instance, Reis et al. (2015) formulated cassava-starch-based films incorporating mango and yerba mate extract, resulting in tensile strength values ranging from 1.36 to 4.03 MPa and elongation values falling within the range of 55 to 69%. It is important to note that the fruit pulp content utilized in that study was at most 20% [26], a value much lower than the 71% juice content used in this work.

### 3.3. Effect of Pear Juice Concentration on the Transparency and Color Attributes of Edible Films

Transparency and homogeneity are critical attributes in the development of food packaging materials [39,40,41]. Films prepared with the purees of fruits and vegetables may contain remaining insoluble fractions that affect their optical properties. Therefore, in this study, it was hypothesized that the use of pear juice instead of the fruit puree could enhance these film characteristics, rendering them more attractive for see-through packaging applications. The effect of pear juice concentration on the transparency and color attributes of edible films containing pregelatinized cassava starch was evaluated. The mid-ripe juice was chosen for this purpose, as it allowed the production of films that were the easiest to handle and that exhibited the best mechanical properties (Section 3.2).

The color attributes and transparency of films containing different concentrations of pear juice (26 wt.%, 46 wt.%, 71 wt.%, and 96 wt.%.) are shown in Table 3. Additionally, for comparative purposes, these attributes are also provided for film samples containing a puree concentration of 26 wt.%, a value commonly used in the formulation of such films [4,8]. It is noteworthy that the films containing pregelatinized cassava starch and 26 wt.%, 46 wt.%, and 71 wt.% pear juice all showed good handling characteristics. These films were dense and continuous, demonstrating good structural integrity and the capability to be properly detached from the cast surface. However, the film with the highest juice content (96 wt.%) exhibited excessive adhesiveness, making it challenging to peel off from the cast surface at room temperature, a behavior probably explained by the relatively high content of sugars in the formulation.

All films made from pear juice were homogeneous and showed high transparency, with similar values regardless of the juice concentration used. In addition, all the films displayed a reddish-yellowish coloration, as indicated by the positive values of a* and b*, which is associated with the enzymatic browning of the initial juice incorporated in the formulations. Accordingly, the increase in the juice content led to higher values of a* and b* and lower values of lightness (L*). This effect was particularly pronounced in the film containing 96 wt.% pear juice. Conversely, no significant differences in L*, a*, and b* values were observed between films with 46 and 71 wt.% juice concentrations. Previous contributions have already reported a decrease in the L* parameter and an increase in a* and b* values with increasing fruit content [23]. Regarding the film made from puree (using the classical 26 wt.% formulation), it showed a very rough and heterogeneous surface, resulting in a lower lightness value than that of the film made with 26 wt.% juice, as well as significantly lower transparency than all juice-based formulations (Table 3).

Recognizing the significance of transparency and homogeneity in films intended for use as packaging materials [52,53,54], the following assays were performed with formulations involving pear juice rather than complete fruit puree. In addition, aiming to maximize the content of film-forming carbohydrates, nutrients, and antioxidants derived from pear, the highest concentration of juice that resulted in a continuous film that could be easily detached from the cast surface was chosen. In this context, the films with 71 wt.% mid-ripe pear juice, 0.8 wt.% glycerol, and 3 wt.% pregelatinized cassava starch were chosen for further characterization in comparison with the corresponding films without starch addition.

### 3.4. Optical Properties, Moisture Content, Water Solubility, Contact Angle, Swelling, and Water Vapor Permeability of the Films

Table 4 summarizes the results concerning the optical properties of the films. It can be seen that the addition of pregelatinized cassava starch does not affect the transparency of the films. However, it induces changes in their color attributes when compared with the films without starch (ΔE~19), with a marked increase in their lightness and a decrease in their redness (a*) and yellowness (b*) values. Similar effects were previously reported in papaya films upon gelatin addition [24]. Dalle Mulle Santos et al. (2016) also observed a similar behavior (increased L* and reduced a* values) when gelatinized cassava starch was added to film-forming formulations based on avocado oil extraction residue containing 0.5 and 1.0 wt.% glycerol. The authors attributed the changes in L* and a* values to the dilution of the original solution with starch (whose films often have high luminosity, >80%, and low a* and b* absolute values [2]), thereby leading to a lighter color spectrum and a decrease in the color intensity of the samples [22].

The water sensitivity of the films is also evidenced in Table 4. Concerning their moisture content, both films (i.e., with and without pregelatinized cassava starch) exhibited values close to 17%, aligning with the moisture contents frequently reported for fruit or vegetable puree-based films [7,20,22,26,55]. The addition of pregelatinized cassava starch did not significantly impact the moisture content of the films.

In terms of water solubility, the pear juice film showed high solubility (87.7 ± 3.9%). Wet films were difficult to recover and rapidly disintegrated with agitation or vortexing. The addition to 3 wt.% of pregelatinized cassava starch led to films with lower water solubility (*p* < 0.05), which were much less prone to disintegration after wetting. Silva et al. (2018) stated that the addition of hydrophilic constituents does not always lead to the higher solubility of films. The type of bond, its strength, and charge of film constituents also influence the film solubility [55,56]. In Table 4, the reduction in the water solubility of films containing pregelatinized cassava starch suggests that the addition of this biopolymer facilitated additional hydrogen bonding interactions with juice components (e.g., pectin, sugars, polyphenols), reducing the availability of their hydroxyl groups to interact with water [57]. Interactions between starch and pectin have been proposed to occur mainly through hydrogen bonds, with pectin molecules interacting with starch mainly through side chains [58]. Previous contributions dealing with films made from starch and fruit purees have also linked the reduction in the water solubility of the films to the interaction of hydrophilic fruit components and the hydroxyl groups of starch [20,59]. The water solubility values shown in Table 4 are consistent with previous results reported for films with high contents of fruit puree and different binding agents (pectin, sodium alginate, gelatin, starch, 1–5 wt.%) [20,24,55]. Films with high solubility in water are advantageous for packaging portions of food ingredients or additives intended to be dispersed in water or food mixtures (individual soup, instant drinks, sugar, and spice portions) [7]. Furthermore, they have been proposed as packaging for fatty foods and oils [4,25,60]. For instance, films based on cassava starch, and mango and acerola fruit pulps (0–20%) were employed to pack and protect palm oil from oxidation. The authors’ findings indicate that these films served as an effective barrier against oxidation compared with LDPE films [25].

Contact angle measurements were performed to assess the surface wettability of the developed films. Films made only from pear juice exhibited a contact angle at time zero of 53.8 ± 1.3°. The addition of pregelatinized cassava starch led to a statistically significant decrease in the wettability of the films, with a contact angle of 66.3 ± 2.5°. Both the initial contact angle values fall within the range of values previously reported for films prepared from papaya puree, gelatin, and defatted soy protein [24], as well as for films based on kale puree and sodium alginate [7].

Changes in contact angle values after 150 s, as shown in Table 4, give further evidence of the impact of starch addition on the water sensitivity of the films. Whereas the contact angle of the juice film is reduced by 65.2 ± 3.0%, the contact angle of the film formulated with pregelatinized cassava starch shows a reduction of 25.02 ± 4.6%. In accordance with the solubility values, the initial contact angles and their reduction after 150 s indicate a lower affinity for water in films containing starch, resulting in reduced wettability and a slower water absorption rate. Both results align with a structure in which there are fewer free hydroxyl groups available for interacting with water.

In line with the previous results, the addition of pregelatinized cassava starch to pear juice films also led to a significant reduction in their swelling, with values approximately 50% lower than those for the control films. This further confirms the reduced affinity for water of the films upon starch addition.

In terms of the moisture barrier properties of the films, the values reported in Table 4 are within the range of those reported for other films based on fruits and vegetables (e.g., acerola, banana, cashew apple, guava, mango, papaya, pear, pequi, strawberry, carrot, tomato, and avocado), with water vapor permeability values ranging from 1.66 × 10^−11^ to 3.78 × 10^−9^ g m/(m^2^ s Pa) [4,8,22,61]. In this contribution, the addition of starch led to a slight reduction in the water vapor permeability of the pear juice films. In accordance with previous results, the enhanced moisture barrier property of the films with pregelatinized starch may be associated with their lower hydrophilicity, resulting from fewer free hydroxyl groups available to interact with water. Although there are other factors influencing water vapor permeability, less hydrophilic edible films have been previously associated with materials less permeable to water vapor [62]. Furthermore, the addition of pregelatinized cassava starch might have contributed to the formation of a denser structure, reducing water diffusion through the more closely packed biopolymer network [63].

### 3.5. FTIR Spectra of the Films

**Figure 3:**
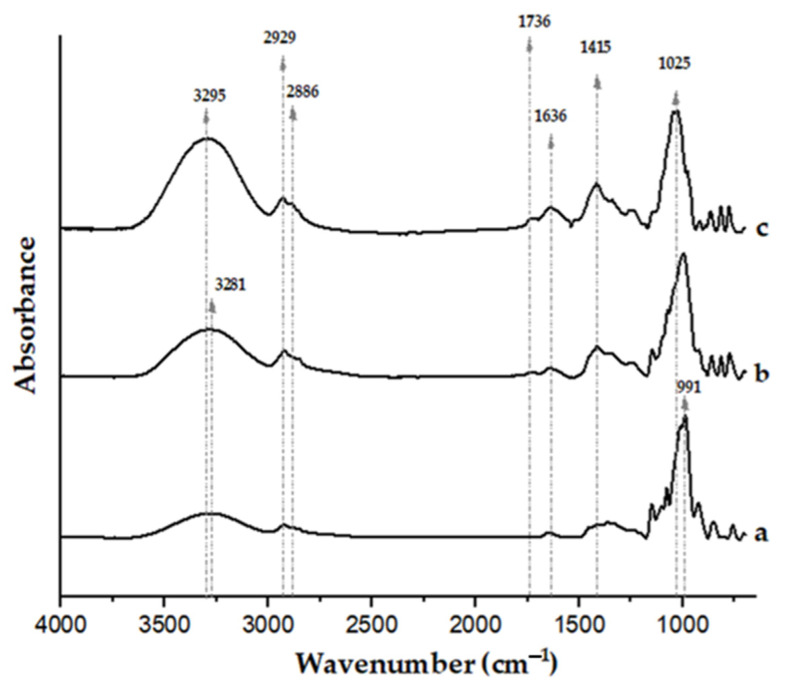
FTIR spectra of a pregelatinized cassava starch film (**a**), film based on mid-ripe pear juice (71 wt.%) and 3 wt.% pregelatinized cassava starch (**b**), and film based on mid-ripe pear juice (71 wt.%) without pregelatinized cassava starch (**c**). All the films contained 0.8 wt.% glycerol.

Figure 3 shows the FTIR spectra of the films. The spectrum of a film made only from pregelatinized cassava starch was also included for comparison.

The spectrum of the film from pear juice (without starch addition, and with 0.8 wt.% glycerol; Figure 3c) shows intense bands in the 900–1100 cm^−1^ range, attributed to the C-O and C-C stretching vibrational modes of carbohydrates present in the fruit [64,65,66]. Signals in the 1400–1450 cm^−1^ region have been associated with the stretching vibrations of methyl esters and free carboxyl groups of pectin [67]. Moisture remaining in the films, even after conditioning, is indicated by the absorbance centered at ≈1636 cm^−1^. This broad band may also have contributions from glycerol groups [68] and the free carboxyl groups of pectin, whose anti-symmetric stretching has been associated with bands centered at 1595–1605 cm^−1^ [67]. The absorbances of the benzene skeleton of polyphenols (1610 cm^−1^, C=C) present in the juice may have also contributed to this broad band [69]. The signal centered at 1736 cm^−1^ is associated with the carbonyl groups of pectin esters [65]. Finally, the absorbances close to 2900 cm^−1^ are attributed to C-H stretching vibrations, whereas the broad band between 3000 and 3600 cm^−1^ corresponds to the stretching vibrations of free and intermolecular-bonded hydroxyl groups of carbohydrates present in the juice and also to glycerol [70].

Upon addition of pregelatinized cassava starch, the FTIR spectrum of the film (Figure 3b) reveals a noticeable reduction of the absorbance associated with OH stretching (3000–3800 cm^−1^) compared with the control juice film, as well as a slight shift of the band to lower wavenumber values. Hydrogen bonding is known to cause the OH peak position to shift to lower wavenumbers [71,72,73]. These observations are consistent with additional hydrogen-bonding interactions between starch and juice components, reducing the availability of hydroxyl groups, as previously inferred from the changes in the water sensitivity and mechanical properties of the films upon incorporation of pregelatinized cassava starch. Previous contributions dealing with films based on starch and different fruit components have also linked shifts to lower wavenumbers in the position of the O-H stretching vibration absorbance (3000–3600 cm^−1^) to greater interactions through hydrogen bonding [74,75,76,77]. Noticeable shifts in the 950–1050 cm^−1^ region to lower wavenumbers upon addition of pregelatinized cassava starch also suggest an increase in molecular interactions due to the incorporation of the binding agent [78].

### 3.6. Total Polyphenol Content and Antioxidant Properties of the Films

Fruit purees and juices have been used as active agents in the development of antioxidant packaging films. Such films have demonstrated the ability to reduce the presence of reactive oxygen species inside the packaging or to release antioxidant compounds into the food product or the surrounding headspace [79]. In particular, previous investigations into the polyphenol composition of pear fruit and juice have revealed the presence of different phenolic compounds such as chlorogenic acid, epicatechin, and catechin in pear fruits of the cultivar (cv) “Williams” [80,81]. However, there are no previous studies dealing with the polyphenol content and the antioxidant activity of edible films containing pear juice.

Table 5 shows the total polyphenol content and the antioxidant properties of the films obtained from mid-ripe pear juice (71 wt.%) with and without the addition of pregelatinized cassava starch (3 wt.%). The resulting films showed much higher total polyphenol content than those reported by Susmitha et al. for corn-starch–gelatin-based edible films incorporated with 5–15% mango (puree and peel) and pineapple pomace (1.3–2.8 mg GAE/100 g) [20]. In the mentioned contribution, the authors attributed the low phenolic content measured in the films to the detrimental effect of the high processing temperatures (75 °C) used during the preparation of the film-forming solution (starch gelatinization and gelatin dissolution). Furthermore, the films produced in the current contribution showed antioxidant activity values similar to those reported by Genskowsky et al. (2015) for chitosan edible films incorporated with maqui berry (210–280 mg Trolox equivalents/100 g film) [82] and Otoni (2017) for films based on peach puree (250–290 mg Trolox equivalents/100 g film) [4].

On the other hand, the films with pregelatinized starch showed a reduction of around 18% in the total polyphenol content compared with the films without starch. As expected, as the total polyphenol content decreases, the antioxidant activity measured by both DPPH and FRAP assays also decreases (Table 5). This behavior aligns with the FTIR results indicating interactions between starch and juice components. Chen et al. (2023) suggested that structural characteristics of phenolic compounds affected their interaction affinity and combination degree with starch molecules [83]. In addition, different authors have reported that several polyphenols can form interactions with starch molecules through hydrogen bonds, hydrophobic interactions, and electrostatic or ionic interactions, which can lead to a decrease in the antioxidant content and activity [80,81,83]. Finally, differences in film solubility (Table 4) may have also played a role in the polyphenol content and antioxidant activities quantified in the aqueous medium used in their determination.

## 4. Conclusions

In the current contribution, the suitability of using pregelatinized cassava starch for the non-thermal production of novel pear juice-based films with antioxidant activity was demonstrated. Continuous films with good structural integrity, which could be properly detached from the cast surface, were obtained in a process completely performed at room temperature. Pregelatinized cassava starch played a key role in increasing the cohesiveness and lightness of the pear juice films while reducing their redness and yellowness, as well as their sensitivity to water. The latter was demonstrated by significantly lower values of water solubility, wettability, water absorption rate, swelling, and water vapor permeability. All the results provided evidence of effective hydrogen-bonding interactions between hydrophilic pear juice components and the hydroxyl groups of starch and glycerol.

On the other hand, the use of pear juice instead of the full puree—the raw material typically used in this type of fruit and vegetable-based films—proved to be highly beneficial for their transparency. In fact, films with suitable transparency that were easy to peel off and handle could be obtained using up to 71 wt.% of pear juice, a value comparatively high considering the typical concentrations of fruit puree used in most contributions (i.e., ≈26 wt.%). The use of such high fruit juice contents resulted in films with elevated total polyphenol content and appealing antioxidant properties. Finally, the findings of the current contribution underscore the importance of considering the degree of ripeness of the fruits used as the raw material for the film-forming formulation. The evolution of the carbohydrate composition in fruits influences the films’ properties, leading to significant variations in their integrity, cohesiveness, and mechanical properties.

Overall, this study suggests that the use of pregelatinized starches in combination with fruit juices may be an interesting alternative for the non-thermal development of highly transparent cohesive edible films with suitable antioxidant activity.

## Figures and Tables

**Table 1:** Yields and monosaccharide composition fractions obtained from the juice of unripe, mid-ripe, and over-ripe pears.

Degree of Ripeness of Pears	MW Fraction ^a^	Yield ^b^ (%)	Composition of Monosaccharides (g/kg)							
			Rha	Ara	Xyl	Man	Gal	Glc	UA	Total
unripe	Low	85.0	-	9	5	1	-	465	55	540
mid-ripe		84.2	-	62	2	-	-	397	78	539
over-ripe		86.7	-	-	15	4	-	532	74	620
unripe	Medium	8.4	3	15	12	6	2	237	38	314
mid-ripe		9.3	1	13	6	5	2	158	32	217
over-ripe		9.6	3	16	7	5	3	235	37	307
unripe	High	6.6	5	96	9	7	30	30	383	560
mid-ripe		6.4	6	187	14	2	16	51	278	554
over-ripe		3.8	18	324	16	5	33	39	189	624

^a^ Low MW: dialyzed through dialysis tubing of MWCO 3500 Da; medium MW: dialyzed through dialysis tubing of MWCO 12–14,000 Da but was retained by dialysis tubing of MWCO 6–8000 Da; high MW: was retained by dialysis tubing of MWCO 12–14,000 Da. ^b^ As percentages of the lyophilized pear juice.

**Table 2:** Mechanical properties of films based on the juice (71 wt.%) from unripe, mid-ripe, and over-ripe pears. In all cases, the films contained 0.8 wt.% glycerol and 3 wt.% pregelatinized cassava starch.

	Young’s Modulus (MPa)	Tensile Strength (MPa)	Strain at Break (%)
Film from unripe pear juice	0.45 ± 0.11 ^a^	0.34 ± 0.06 ^a^	98.0 ± 1.5 ^a^
Film from mid-ripe pear juice	2.61 ± 0.68 ^b^	0.61 ± 0.07 ^b^	57.8 ± 3.9 ^b^
Film from over-ripe pear juice	0.56 ± 0.07 ^a^	0.28 ± 0.04 ^a^	64.7 ± 5.0 ^b^

Different letters in the same column denote statistically significant differences (*p* < 0.05, Games–Howell test).

**Table 3:** Photographs, color attributes, and transparency of films made with mid-ripe pear puree or juice at different juice concentrations. In all cases, the films contain 0.8 wt.% glycerol and 3 wt.% pregelatinized cassava starch.

	Film from Pear Puree, 26 wt.%	Film from Pear Juice, 26 wt.%	Film from Pear Juice, 46 wt.%	Film from Pear Juice, 71 wt.%	Film from Pear Juice, 96 wt.%
	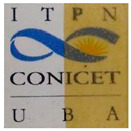	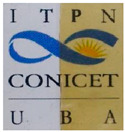	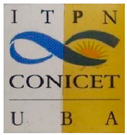	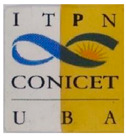	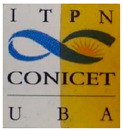
L*	70.8 ± 1.5 ^a^	78.0 ± 1.7 ^b^	72.74 ± 0.82 ^c^	72.0 ± 1.5 ^a,c^	66.6 ± 2.7 ^d^
a*	6.25 ± 0.62 ^a^	3.70 ± 0.65 ^b^	6.59 ± 0.49 ^a^	6.97 ± 0.93 ^a^	11.8 ± 1.7 ^c^
b*	26.7 ± 1.6 ^a^	19.6 ± 2.4 ^b^	29.37 ± 0.95 ^c^	31.1 ± 2.7 ^c^	37.9 ± 2.7 ^d^
Transparency	1.6 ± 0.02 ^a^	4.97 ± 0.10 ^b^	4.86 ± 0.16 ^b^	4.92 ± 0.30 ^b^	4.89 ± 0.25 ^b^

Different letters in the same row denote statistically significant differences (*p* < 0.05, Games–Howell test).

**Table 4:** Moisture content, water solubility (%), contact angle, swelling, and water vapor permeability of films obtained from mid-ripe pear juice (71 wt.%) with and without the addition of pregelatinized cassava starch (3 wt.%). Both films contained 0.8 wt.% glycerol.

Property	Film without Pregelatinized Cassava Starch	Film with 3 wt.% Pregelatinized Cassava Starch
L*	58.6 ± 1.4 ^a^	72.0 ± 1.5 ^b^
a*	15.94 ± 1.3 ^a^	6.97 ± 0.93 ^b^
b*	41.67 ± 0.6 ^a^	31.1 ± 2.7 ^b^
Transparency	4.96 ± 0.40 ^a^	4.92 ± 0.30 ^a^
Moisture content (%)	16.9 ± 1.5 ^a^	17.2 ± 1.5 ^a^
Water solubility (%)	87.7 ± 3.9 ^a^	71.80 ± 0.64 ^b^
Contact angle at 0 s (°)	53.8 ± 1.3 ^a^	66.3 ± 2.5 ^b^
Contact angle at 150 s (°)	18.7 ± 1.2 ^a^	49.7 ± 1.9 ^b^
Swelling degree	12.94 ± 0.97 ^a^	6.49 ± 0.50 ^b^
Water vapor permeability (g m/(m^2^ s Pa))	2.1 ± 0.13 × 10^−10 a^	1.7 ± 0.11 × 10^−10 b^

Different letters in the same row denote statistically significant differences (*p* < 0.05, Student’s *t* test).

**Table 5:** Total polyphenol content and antioxidant activity of films obtained from mid-ripe pear juice (71 wt.%) with and without the addition of pregelatinized cassava starch (3 wt.%). Both films contained 0.8 wt.% glycerol.

Property	Film without Pregelatinized Cassava Starch	Film with 3 wt.% Pregelatinized Cassava Starch
Total polyphenol content (mg GAE/100 g)	700.3 ± 3.8 ^a^	577 ± 23 ^b^
DPPH (mg Trolox equivalents/100 g)	286 ± 20 ^a^	213.7 ± 5.73 ^b^
FRAP (mg FeSO_4_/100 g)	38.07 ± 0.06 ^a^	28.07 ± 0.543 ^b^

Different letters in the same row denote statistically significant differences (*p* < 0.05, Student’s *t* test).

## Data Availability

The data presented in this study are available on request from the corresponding author.
